# Ventriculoatrial malalignment in atrioventricular septal defect resulting in uniatrial biventricular connection: surgical options

**DOI:** 10.1186/s13019-020-01099-x

**Published:** 2020-04-15

**Authors:** Michael Hofbeck, Gesa Wiegand, Jörg Michel, Christian Schlensak

**Affiliations:** 1 Department of Paediatric Cardiology, University Children’s Hospital, Hoppe-Seyler-Str. 1, 72076 Tuebingen, Germany; 2 Department of Thoracic and Cardiovascular Surgery, University Hospital, Hoppe-Seyler-Str. 1, 72076 Tuebingen, Germany

**Keywords:** Uniatrial biventricular connection, Atrioventricular septal defect, Ventriculoatrial malalignment, Echocardiography, Congenital heart disease

## Abstract

**Background:**

Uniatrial biventricular connection (UBC) is a rare cardiovascular anomaly characterized by absence of one atrioventricular connection and drainage of the other atrium via a solitary atrioventricular valve into both ventricles. The absent atrioventricular connection may affect either the left or right atrium. Because of the absence of one atrioventricular connection hearts with UBC have been classified among functionally univentricular hearts requiring palliative treatment according to the Fontan principle.

**Aims/objective:**

We report two further patients with UBC. In one of these patients careful echocardiographic examination of the atrioventricular junction in early infancy revealed the possibility of biventricular repair based on the favorable anatomy of the atrioventricular valve and balanced ventricles in the presence of an inlet ventricular septal defect (VSD).

**Case presentations:**

Both patients presented in the neonatal period for evaluation of complex congenital heart disease. The anatomy of the atrioventricular valves in our patients was indistinguishable from atrioventricular septal defects exhibiting the morphology of a common valve with superior and inferior bridging leaflets. The common atrioventricular valve was connected exclusively to the right atrium draining into both ventricles while the left atrium drained into the right atrium via a secundum atrial septal defect. In one of our patients biventricular repair with good longterm-result was performed by reseptation of the atria, patch repair of the VSD and septation of the atrioventricular valve. The second patient underwent univentricular palliation according to the Fontan principle.

**Discussion/conclusion:**

The echocardiographic findings in our patients suggest that at least some patients with UBC represent a variant of atrioventricular septal defects associated with extreme ventriculoatrial malalignment resulting in fusion of the deviated primary atrial septum with the lateral aspect of the atrioventricular junction. This offers the option of septation of the common atrioventricular valve and biventricular repair in patients with adequate size of both ventricles. Exact echocardiographic analysis of the morphology of the atrioventricular valve is essential to distinguish these patients with a morphologically common atrioventricular valve in early infancy from other variants of absent atrioventricular connection and to select those who are suitable for biventricular repair.

## Background/introduction

Hearts with UBC are characterized by the absence of one atrioventricular connection while the other atrium drains into both ventricles [1, 2]. The absent atrioventricular connection may affect either the left or right atrium. Since the contralateral atrium is draining into both ventricles, these hearts also belong to the spectrum of double outlet right atrium or double outlet left atrium [1, 3, 4].

Because of the absence of one atrioventricular connection hearts with UBC have been classified among functionally univentricular hearts [2]. In our paper we report two further patients. Based on the favorable anatomy of the atrioventricular valve and balanced ventricles in the presence of an inlet ventricular septal defect (VSD) one of these patients underwent successful biventricular repair.

## Case presentations

**Fig. 1:**
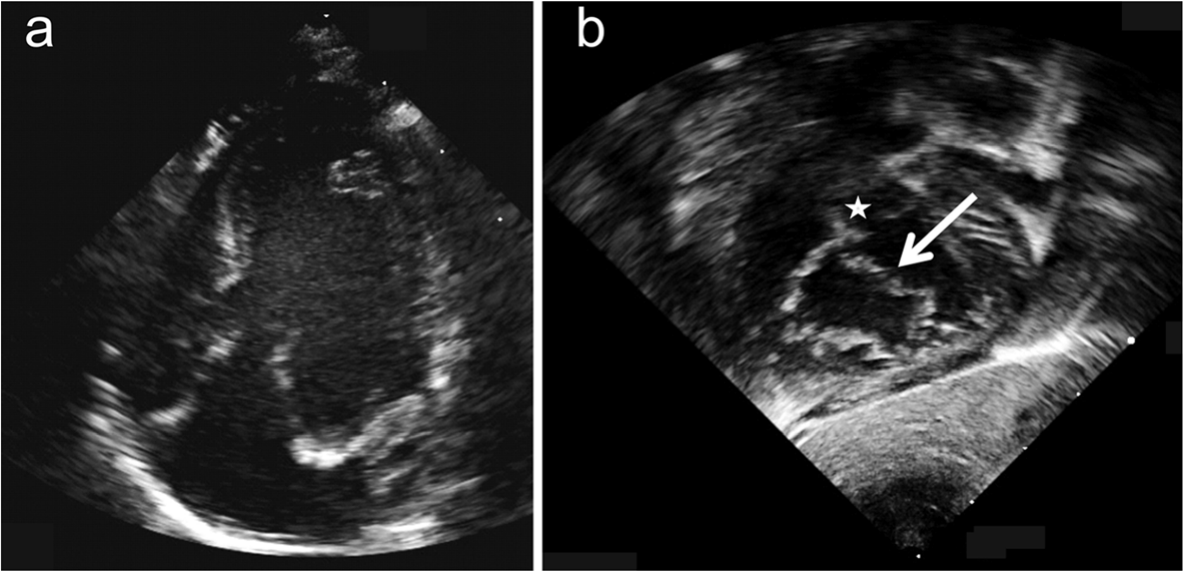
Apical 4-chamber view in patient 1 shows absent left atrioventricular connection, UBC, hypoplastic right ventricle and a solitary atrioventricular valve straddling a large inlet VSD (**a**). The subcostal LAO-view (**b**) shows the morphology of a common atrioventricular valve with a superior bridging leaflet (arrow) inserting to the right of the ventricular septum (asterix)

Our first patient was born at term. Echocardiography in the newborn period showed situs solitus, levocardia and absent left atrioventricular connection. The right atrium drained via a solitary atrioventricular valve into both ventricles. The morphologic left ventricle was dominant while the right ventricle was significantly hypoplastic (Fig. 1a). Both ventricles communicated via a large inlet ventricular septal defect. The atrioventricular valve had striking similarity to the common atrioventricular valve in patients with atrioventricular septal defect displaying superior and inferior bridging leaflets (Fig. 1b). Both atria were connected by an unrestrictive secundum atrial septal defect. Ventriculoarterial connections were normal. In addition the child had valvular pulmonary stenosis and a left superior vena cava draining to the coronary sinus. Right ventricular hypoplasia precluded biventricular repair. At the age of 2 months she underwent placement of a modified right Blalock-Taussig shunt and transsection and closure of the main pulmonary artery followed by creation of a bilateral bidirectional Glenn anastomosis at the age of 6 months. Following completion of the Fontan circulation performed with an extracardiac tunnel at the age of 3.4 years the patient is doing well at the age of 6 years.

**Fig. 2:**
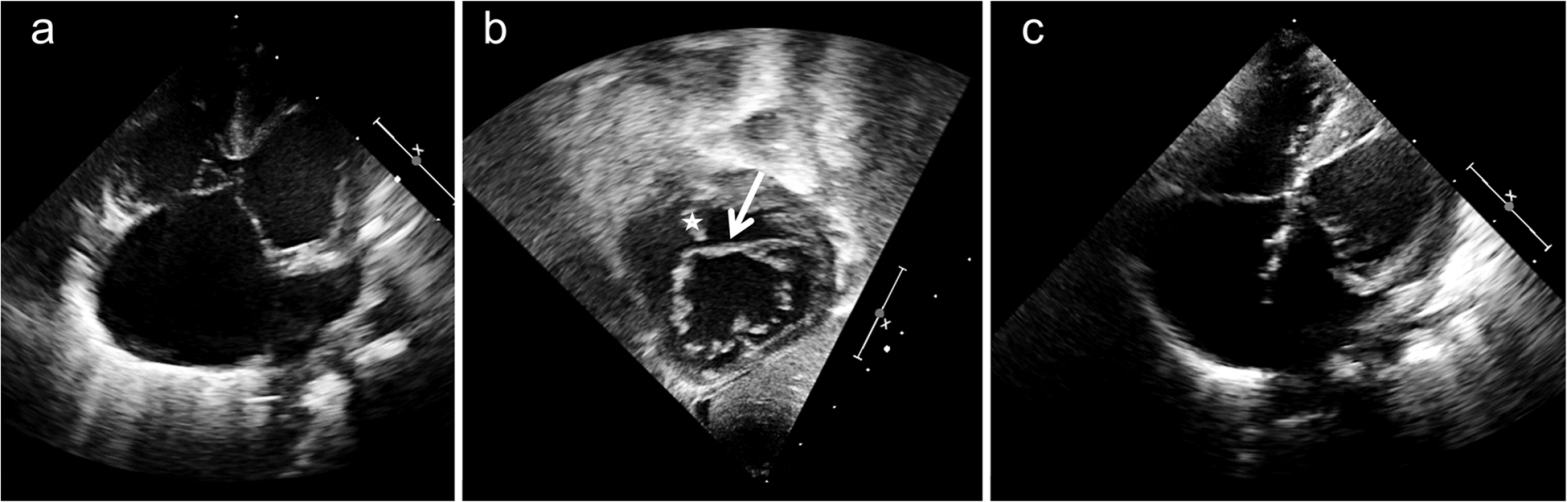
In patient 2 the apical 4-chamber view demonstrates UBC with absent left atrioventricular connection and balanced ventricles (**a**) (Video 1). The subcostal LAO view reveals a common atrioventricular valve with superior (arrow) and inferior bridging leaflets (**b**) (Video 2). The ventricular septum is marked by an asterix. Following surgery the apical 4-chamber shows successful septation of the common atrioventricular valve (**c**)

Our second patient presented at the age of 8 days with severe congestive heart failure. Echocardiography revealed situs solitus, levocardia and absent left atrioventricular connection (Fig. 2a, Video 1). The atrioventricular valve was connected to the right atrium and drained into both ventricles which were both of adequate size. The atrioventricular valve had the features of a common atrioventricular valve indistinguishable from patients with atrioventricular septal defect exhibiting superior and inferior bridging leaflets (Fig. 2b, Video 2). A large inlet VSD connected both ventricles. Color Doppler revealed no significant atrioventricular valve regurgitation and no primum ASD. The inferior portion of the atrial septum was connected to the left lateral margin of the atrioventricular junction. The left atrium drained via a nonrestrictive secundum ASD. Ventriculoarterial connections were normal and without obstruction. The patient underwent palliative pulmonary artery banding at the age of 3 weeks. Biventricular repair and debanding of the pulmonary artery were performed at the age of 2 months. At surgery the common atrioventricular valve presented an inferior bridging leaflet adherent to the crest of the ventricular septum while there was a large VSD underneath the superior bridging leaflet. The superior bridging leaflet straddled the ventricular septum inserting in a papillary muscle at the right side of the septum (equivalent to Rastelli type B of common atrioventricular canal) [5]. The inlet ventricular septal defect was closed by a patch separating the atrioventricular valve into two orifices. Following excision of the atrial septum the atria were reseptated with a pericardial patch. Because of the relatively small size of the left atrioventricular valve the apposition zone (so-called “cleft”) between the superior and inferior brigding leaflet was only partially closed. The postoperative course was uneventful. Follow-up examinations revealed unobstructed venous flow from both atria and only mild left-sided atrioventricular valve insufficiency (Fig. 2c). At the age of 9 years the patient is in excellent clinical condition.

## Discussion/conclusion

Hearts with UBC represent a rare but distinct cardiovascular anomaly [2]. The largest series was published by Kiraly et al. who collected a total of 14 postmortem specimens from 3 institutions [2]. In this study 11 hearts showed absent right while 3 had absent left atrioventricular connection. The straddling atrioventricular valve connecting one atrium to both ventricular chambers had striking similarity to the common atrioventricular valve of patients with atrioventricular septal defect [2]. The authors of this study however suggested not to group UBC among atrioventricular septal defects (AVSD): They argued that a common atrioventricular valve characterizing AVSD is an anatomic impossibility in patients with absence of one atrioventricular connection.

**Fig. 3:**
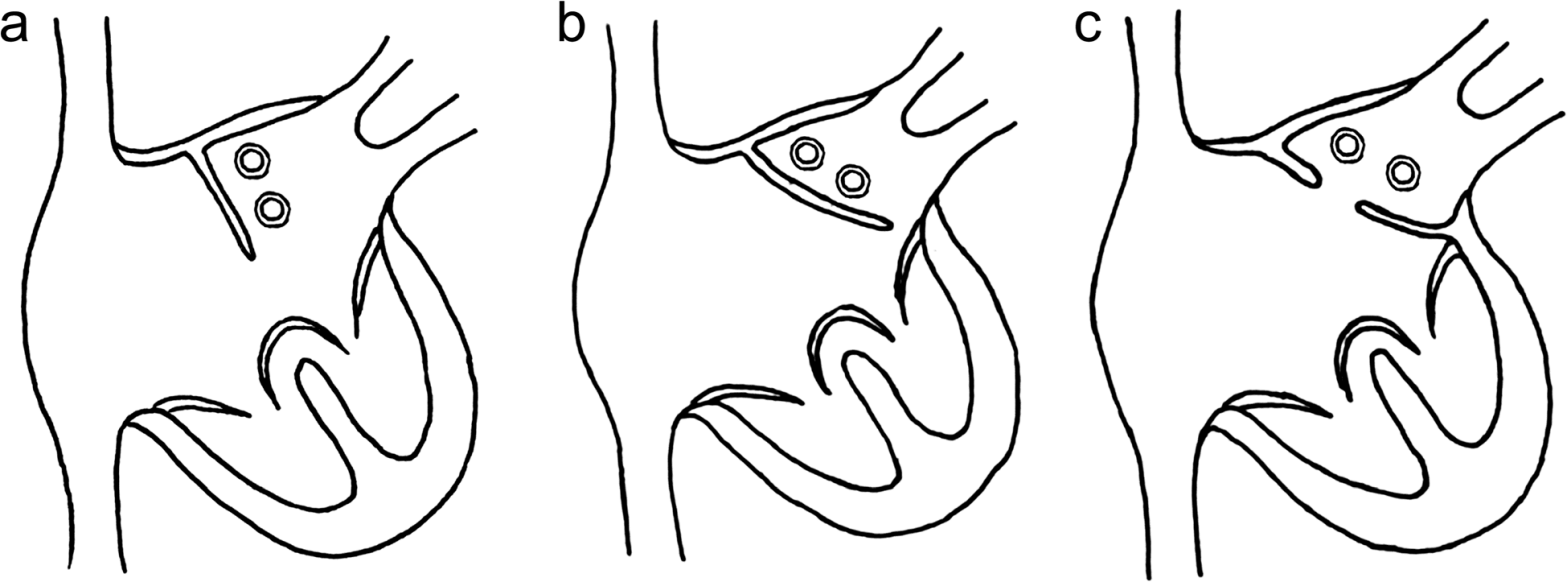
AVSD with normal orientation of the atrial septum (**a**). Atrioventricular malalignment with leftward deviation of the atrial septum results in double outlet right atrium (**b**). Extreme atrioventricular malalignment and fusion of the septum primum with the lateral atrioventricular junction results in UBC (**c**). (Redrawn according to [6, 7])

Based on the experience in our patients and on previous reports in the literature we believe that the paradox of one absent atrioventricular connection in the presence of an AVSD does exist. It can be explained as the result of extreme ventriculoatrial malalignment (Fig. 3): It is well known, that ventriculoatrial malalignment in a subgroup of patients with AVSD may result in significant leftward or rightward deviation of the atrial septum [6, 8]. In this situation one atrium drains via the primum ASD while the other atrium is connected via the common atrioventricular valve to both ventricles representing double outlet right or left atrium [3, 8, 9]. In the majority of cases these patients present with partial AVSD, leftward deviation of the atrial septum and intact ventricular septum [6, 7, 10, 11]. Pronounced ventriculoatrial malalignment may even result in diminution in size of the primum ASD with obstruction of the respective atrial outlet and subsequent development of postcapillary pulmonary hypertension [6, 12, 13]. Extreme malalignment with fusion of the primary atrial septum with the lateral aspect of the atrioventricular junction results in obliteration of the primum ASD, absence of one atrioventricular connection and UBC (Fig. 3c). However the morphology of the atrioventricular valve appears not to be influenced by this malalignment. This explains the striking similarity of the atrioventricular valve between patients with AVSD and at least some of the patients with UBC. These characteristics include the presence of superior and inferior bridging leaflets with a line of coaptation perpendicular to the plane of the ventricular septum and the presence of 4 or 5 valve leaflets [2]. These morphologic features were evident echocardiographically in both of our patients and confirmed by the intraoperative findings in our patient who underwent biventricular repair.

It has been suggested to group all patients with UBC among functionally univentricular hearts requiring univentricular palliation since biventricular repair appeared impossible in the presence of a unilateral absent atrioventricular connection [2]. However there are several reports in the literature describing biventricular repair in patients with UBC: In our second patient the morphology of the atrioventricular valve corresponded with type B of the Rastelli classification for common atrioventricular canal, exhibiting superior and inferior bridging leaflets with a large VSD located underneath the superior bridging leaflet [5]. Following excision of the atrial septum the AVSD was corrected by double patch technique. Westerman et al. reported successful biventricular repair in a patient with UBC, common atrioventricular valve and tetralogy of Fallot [14]. The atrial septum deviated to the left resulting in absence of a primum ASD, absent left atrioventricular connection and double outlet right atrium. The morphology of the common atrioventricular valve was classified as Rastelli type C [5]. Horiuchi et al. described successful biventricular repair in another patient with UBC, L-loop double outlet right ventricle and pulmonary stenosis [15]. In this patient the right atrioventricular connection was absent and the right atrium drained via a secundum atrial septal defect. The morphology of the atrioventricular valve was described as Rastelli type C [5]. Less detailed description of the anatomy of the atrioventricular valve is provided in the report of Stark et al. describing surgical repair in one patient with complete AVSD and absent left atrioventricular connection who underwent atrial reseptation and repair of the AVSD by double patch technique [16]. Surgical repair in another patient with UBC and partial AVSD was described by Corwin et al. [17]: In their patient with leftward deviation of the atrial septum and absent left atrioventricular connection the left atrium drained via a secundum ASD. The atrioventricular valve presented with two orifices while the ventricular septum was intact. Surgical repair was achieved by excision of the atrial septum and reseptation of the atria [17].

While it has been suggested to address the atrioventricular junction in these patients as solitary and not as common [1], the morphology of the atrioventricular valve offers the possibility of treatment according to the surgical techniques for repair of atrioventricular septal defects. In the preoperative assessment it is essential to discriminate patients with atrioventricular septal defect and ventriculoatrial malalignment from patients with absent atrioventricular connection associated with straddling of the mitral or tricuspid valve [18, 19]. This differentiation is well possible based on transthoracic echocardiography. Characteristic echocardiographic features of AVSD are the presence of a morphologically common atrioventricular valve exhibiting both left and right components as well as superior and inferior bridging leaflets straddling the ventricular septum. While it may be difficult to define this with certainty in the apical 4-chamber view (Video 1), the anatomy of the common atrioventricular valve can be well displayed in the parasternal short axis view and in the subcostal views. Especially the subcostal LAO-view reveals the nature of the common atrioventricular valve with its superior and inferior bridging leaflets (Figs. 1b, 2b, Video 2). However the option of biventricular repair depends not only on the morphology of the atrioventricular valve but also on the presence of two adequately sized ventricles and on the nature of ventriculo-arterial connections. Clarification of the anatomic details is essential during initial assessment in the first weeks of life, since the decision for univentricular palliation versus biventricular repair is made in early infancy.

Echocardiographic evaluation of infants with absent right or left atrioventricular connection should include the differential diagnosis of UBC with a morphologically common atrioventricular valve. Univentricular palliation according to the Fontan principle is associated with significant long-term morbidity. If the presence of the common atrioventricular valve is missed on echocardiography the respective patient may be deprived of the option of possible biventricular repair and its associated better long-term prognosis.

## Supplementary information

**Additional file 1: Video S1.** Apical 4 chamber view in patient 2 showing UBC with absent left atrioventricular connection. Both ventricles are of equal size. Note the atrial septum connecting to the left lateral aspect of the atrioventicular junction and the absence of a primum ASD.

**Additional file 2: Video S2.** The subcostal LAO view in patient 2 reveals a common atrioventricular valve with superior and inferior bridging leaflets.

## Data Availability

Not applicable.

## Abbreviations

ASD: Atrial septal defect
AVSD: Atrioventricular septal defect
UBC: Uniatrial biventricular connection
VSD: Ventricular septal defect

## References

[1] Gupta SK, Gupta A, Ramakrishnan S, Anderson RH (2015). Clarifying the atrioventricular junctional anatomy in the setting of double outlet right atrium. Ann Pediatr Cardiol..

[2] Kiraly L, Hubay M, Cook AC, Ho SY, Anderson RH (2007). Morphologic features of the uniatrial but biventricular atrioventricular connection. J Thorac Cardiovasc Surg.

[3] Praagh RV (2013). What are double-outlet left atrium and double-outlet right atrium?. Ann Pediatr Cardiol.

[4] Shetkar SS, Kothari SS (2013). Double-outlet left atrium: Ventriculo-atrial malalignment defect. Ann Pediatr Cardiol..

[5] Rastelli G, Kirklin JW, Titus JL (1966). Anatomic observations on complete form of persistent common atrioventricular canal with special reference to atrioventricular valves. Mayo Clin Proc.

[6] Brancaccio G, Amodeo A, Rinelli G, Filippelli S, Sanders SP, Di Donato RM (2007). Double-outlet right atrium: anatomic and clinical considerations. Ann Thorac Surg.

[7] Edwin F, Kinsley RH, Mamorare HM, Govendrageloo K (2012). The spectrum of double-outlet right atrium including hearts with three atrioventricular valves. Eur J Cardiothorac Surg.

[8] Anderson RH, Spicer D (2010). Anatomy of common atrioventricular junction with complex associated lesions. World J Pediatr Congenit Heart Surg.

[9] Anderson RH (2013). How best to explain unexpected arrangements of the atrioventricular valves?. Ann Pediatr Cardiol..

[10] Perez-Martinez VM, Garcia-Fernandez F, Oliver-Ruiz J, Nunez-Gonzalez L (1984). Double-outlet right atrium with two atrioventricular valves and left atrial outlet atresia. J Am Coll Cardiol.

[11] Radermecker MA, Chauvaud S, Carpentier A (1995). Double-outlet right atrium with restrictive ostium primum and incomplete supravalvular ring presenting as congenital mitral valve stenosis. J Thorac Cardiovasc Surg.

[12] Ahmadi A, Mocellin R, Spillner G, Gildein HP (1989). Atrioventricular septal defect with double-outlet right atrium. Pediatr Cardiol.

[13] Alivizatos P, Anderson RH, Macartney FJ, Zuberbuhler JR, Stark J (1985). Atrioventricular septal defect with balanced ventricles and malaligned atrial septum: double-outlet right atrium. Report of two cases. J Thorac Cardiovasc Surg.

[14] Westerman GR, Norton JB, Van Devanter SH (1986). Double-outlet right atrium associated with tetralogy of Fallot and common atrioventricular valve. J Thorac Cardiovasc Surg.

[15] Horiuchi T, Saji K, Osuka Y, Sato K, Okada Y (1976). Successful correction of double outlet left atrium associated with complete atrioventricular canal and l-loop double outlet right ventricle with stenosis of the pulmonary artery. J Cardiovasc Surg (Torino).

[16] Starc TJ, Bierman FZ, Bowman FO, Steeg CN, Wang NK, Krongrad E (1987). Pulmonary venous obstruction and atrioventricular canal anomalies: role of cor triatriatum and double outlet right atrium. J Am Coll Cardiol.

[17] Corwin RD, Singh AK, Karlson KE (1983). Double-outlet right atrium: a rare endocardial cushion defect. Am Heart J.

[18] Coto EO, Calabro R, Marsico F, Lopez Arranz JS (1981). Right atrial outlet atresia with straddling left atrioventricular valve. A form of double outlet atrium. Br Heart J.

[19] Ho SY, Milo S, Anderson RH, Macartney FJ, Goodwin A, Becker AE (1982). Straddling atrioventricular valve with absent atrioventricular connection. Report of 10 cases. Br Heart J.

